# Compressive properties of self-healing microcapsule-based cementitious composites subjected to freeze-thaw cycles using acoustic emission

**DOI:** 10.3389/fchem.2022.940184

**Published:** 2022-09-23

**Authors:** Wenfeng Hao, Hao Hao, Humaira Kanwal, Shiping Jiang

**Affiliations:** ^1^ College of Mechanical Engineering, Yangzhou University, Yangzhou, China; ^2^ Faculty of Civil Engineering and Mechanics, Jiangsu University, Zhenjiang, China

**Keywords:** self-healing cementitious composites, freeze-thaw cycles, acoustic emission, compressive properties, microcapsules, frost resistance

## Abstract

Microcapsule self-healing technology is an effective scheme to improve the durability of cementitious composites. In this paper, the compressive properties of microcapsule-based self-healing cementitious composites after freeze-thaw cycles were studied using acoustic emission (AE), and the changes in AE characteristics, compressive strength, mass loss rate, and electric flux of microcapsule-based self-healing cementitious composites with different microcapsule contents and freeze-thaw cycles were studied. The results show that if the content of the microcapsule is appropriate, with the increase in the number of freeze-thaw cycles, the AE hits will generally increase first and then decrease, and the early AE events will also decrease. Because of the different contents of microcapsules, the improvement effect and defect effect change dynamically with the number of freeze-thaw cycles, which is also reflected in the dynamic process of compressive strength. After 100 freeze-thaw cycles, the compressive strength of self-healing cementitious composite samples with 5% content of microcapsules and 3% content of microcapsules is the highest. The changes in mass loss rate and electric flux are similar to the AE characteristic parameters, which further verifies the results of AE. The research results of this paper provide experimental data and experimental methods for the engineering application of microcapsule self-healing cement-based composites in cold areas.

## Introduction

Since White et al. (2001) developed microcapsules with self-healing function, microcapsule self-healing cementitious composites are widely investigated by scholars. After adding microcapsules into cement-based materials, various properties of materials have changed, including workability, permeability, elasticity, and strength. The final effect depends on the dose, size, carrier, and special characteristics of microcapsules. The advantages of microcapsule self-healing cementitious composites are prolonging the service life of concrete, reducing the cost of energy, and improving the service life of concrete (Zhang et al., 2020). Microcapsule-filled self-healing composites have become a research hotspot in the field of self-healing because of their unique advantages (Xu et al., 2021a; Han et al., 2021; Yang et al., 2021; Wang et al., 2022a; Wang et al., 2022b). In the freeze-thaw cycle in cold areas, due to osmotic pressure, temperature stress fatigue failure, and pore water expansion failure, it is easier to lead to internal cracking and surface spalling of concrete. The mechanical properties and self-healing effect of microcapsule-based self-healing cementitious composites after freeze-thaw cycles are worthy of further study.

Frost resistance is one of the important indexes to evaluate the durability of cement-based composites. Scholars have done a lot of research work on the frost resistance of cement-based composites and put forward a series of hypotheses according to the failure mechanism of concrete after the freeze-thaw cycle (Wang et al., 2019; Ma et al., 2021; Huang et al., 2022; Jiang et al., 2022; Zheng et al., 2022). Among these theoretical hypotheses, the pore structure hypothesis, temperature stress fatigue failure hypothesis, hydrostatic pressure hypothesis, and osmotic pressure hypothesis are the most representative. Affan et al. (Affan and Ali, 2022) conducted an experimental investigation on mechanical properties of jute fiber reinforced concrete under freeze-thaw conditions for pavement applications. Zhang et al. (Zhang et al., 2022) studied dynamic and static interfacial bonding properties of CFRP–concrete subjected to freeze-thaw cycles. Du et al. (Du et al., 2022) analyzed the influence of opposite-side high temperature on the frozen behavior of containment concrete under the single-side salt freeze-thaw method.

Acoustic emission (AE) analysis is a sound-based technology, which has been widely used to monitor the crack generation and healing behavior of cement-based materials (Liu et al., 2022). Santosh et al. (Shah and Chandra Kishen, 2010) studied the fracture behavior of concrete interface by using acoustic emission event number and acoustic emission energy, and analyzed the results of load, displacement, CMOD, acoustic emission event, and acoustic emission energy. Zhou et al. (Zhou et al., 2021) studied the acoustic emission characteristics of tailings cemented paste filling under uniaxial compression. The acoustic emission characteristic information reflects the evolution of internal microcracks and the initiation, propagation, and penetration of new microcracks. Xu et al., (2021b) linked the acoustic emission parameters with the mechanical properties of concrete and analyzed the similarities and differences between rubber crumb concrete and ordinary concrete in the whole failure stage.

Although self-healing cement-based composites have been studied, their mechanical properties and healing effect under freeze-thaw cycles are still an open topic. Moreover, the AE characteristics of microcapsule self-healing cement-based composites after freeze-thaw cycles have not been reported. In this paper, the compressive properties of microcapsule-based self-healing cementitious composites under freeze-thaw cycles were studied using AE, and the changes in AE characteristics, compressive strength, mass loss rate, and electric flux of microcapsule-based self-healing cementitious composites with different microcapsule contents and freeze-thaw cycles were studied. The research results of this paper provide experimental data and experimental methods for the engineering application of microcapsule self-healing cement-based composites in cold areas.

## Experimental details

### Preparation of microcapsules

Self-healing microcapsules were prepared by extrusion round and spray drying methods. Firstly, sodium silicate hexahydrate, expansive Portland cement, microcrystalline cellulose, tween 80, and methylcellulose are mixed evenly according to a certain mass ratio, and then appropriate water is added as the core material. The core particles were produced by extrusion spheronization. Finally, the ethyl cellulose solution is sprayed on the capsule core material, and after blast drying, the capsules are dispersed with each other. Ethyl cellulose is a common capsule wall material because of its strong film-forming ability, certain tightness, and certain surface roughness. We mainly control the quality by controlling the particle size. In order to reduce the experimental error, we have screened many times. Firstly, we will fully stir the capsule core material, round and air dry the capsule core, and conduct the first screening to screen the capsule core with a particle size of about 0.9–1 mm. The coating process is to reduce the rotating speed, evenly distribute it near the inner wall of the rounding machine, turn on the rounding machine, spray the coating solution at a uniform speed with a needle tube, and after drying, we conducted a second screening, and finally obtained a 1–1.2 mm microcapsule. In the third selection, we eliminated the microcapsules with obvious defects and tried to ensure the consistency of quality and size. For details of the specific preparation process and relevant raw materials, the previously published papers (Jiang et al., 2021; Wang et al., 2022c) can be referred to.

### Preparation of microcapsule-based self-healing cementitious composites

The preparation method of microcapsule-based self-healing cementitious composites mainly refers to GB/T 17,671–1999 test method for the strength of cement mortar. The sample material is Helin brand ordinary portland cement P.O.42.5R and ISO standard sand, and its size is 40 mm × 40 mm × 40 mm. Microcapsule content refers to the percentage of microcapsule dosage and cement dosage, which are 0%, 1%, 3%, 5%, and 7% respectively in this paper. According to the previous study (Jiang et al., 2021; Wang et al., 2022c), the proportion of sodium fluosilicate is 15% of microcapsules. Since fluosilicate is insoluble in water, sodium fluosilicate is mixed with cement first, and then put into a cement mortar mixer for mixing. In order to avoid the damage of microcapsules during mixing as much as possible, microcapsules are added after the mixing of cement, sand, water, and sodium fluosilicate is completed. After mixing for 1 minute, they are loaded into the mold, vibrated, and cured. The specific preparation process can refer to (Jiang et al., 2021; Wang et al., 2022c). After the prepared samples were cured to a certain age under standard conditions, the changes of strength loss, mass loss, and electric flux of cement-based composites before and after different freeze-thaw cycles were compared to characterize the frost resistance of cement-based composites and the influence of microcapsules on the frost resistance. The freeze-thaw cycle test mainly refers to the Chinese standards test method for long-term performance and durability of ordinary concrete (GB/T 50,082–2009) and the test method for frost resistance of cement mortar. The freeze-thaw cycle test is carried out by a concrete rapid freeze-thaw tester (KDR-V5). Each freeze-thaw cycle shall be completed within 2.5–4 h, and the melting and freeze-thaw time shall not be less than 1.2 h. When freezing and melting are completed, the central temperature of the test piece shall be controlled at -18 ± 2°C and 5 ± 2°C respectively, and the time taken for the test piece to drop from 3°C to −16°C shall not be less than 1/2 of the freezing time. The time taken for the test piece to rise from −16 to 3°C shall not be less than 1/2 of the whole melting time. The temperature difference inside and outside the test piece should not exceed 28°C, and the conversion time between freezing and thawing should not exceed 10 min. Three samples shall be used for each group of tests, the marks shall be made to prevent spalling, and the distribution of test blocks in each sleeve shall be the same to reduce the experimental error. After 25, 50, 75, and 100 cycles respectively, the mass loss test, strength loss test, and electric flux test are carried out. In the compression experiments, AE monitoring is carried out according to the method of literature (Liu et al., 2022).

## Experimental results and discussion

### Compressive strength

**FIGURE 1:**
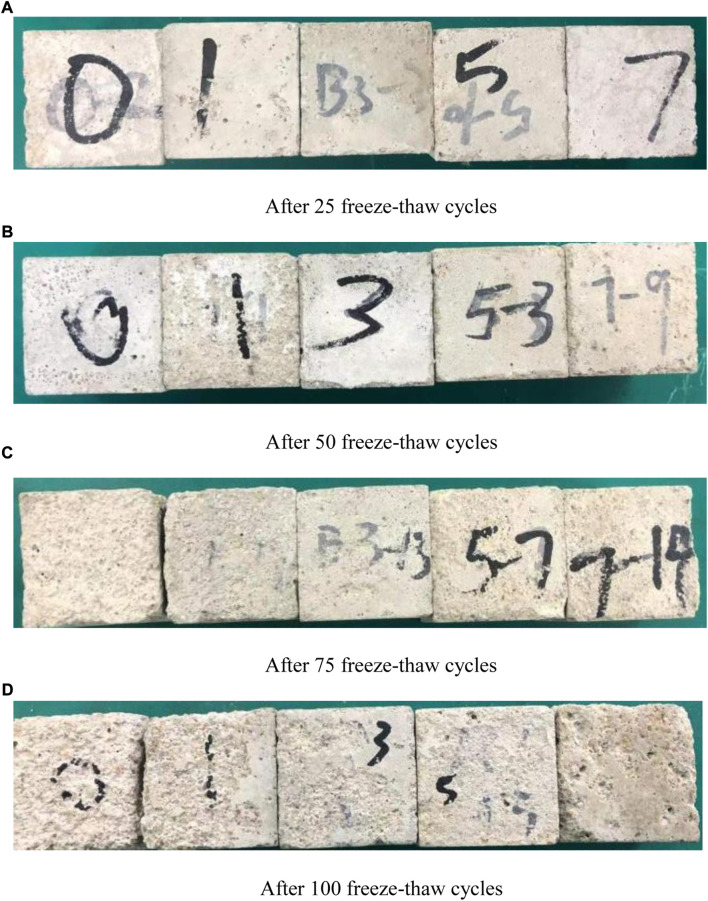
Damage after freeze-thaw cycles. **(A)** After 25 freeze-thaw cycles **(B)** After 50 freeze-thaw cycles **(C)** After 75 freeze-thaw cycles **(D)** After 100 freeze-thaw cycles.

From the freeze-thaw failure test block in Figure 1, it can be found that the microcapsule is triggered when the cement-based composite is subjected to freeze-thaw erosion and spalling. When the ice melts into water, the microcapsules absorb water and expand, and react with the healing agent to produce bonding products. The internal water expansion of cement-based composite test block will lead to crack expansion and external spalling. If microcapsules can play a self-healing role, each freeze-thaw cycle can be regarded as a repair process after damage. It can be seen from Figure 1 that with the increase of self-healing components, the spalling of the test block becomes less and the test block remains more complete. The addition of a self-healing system increases its frost resistance, which is also verified by the change of compressive strength.

**FIGURE 2:**
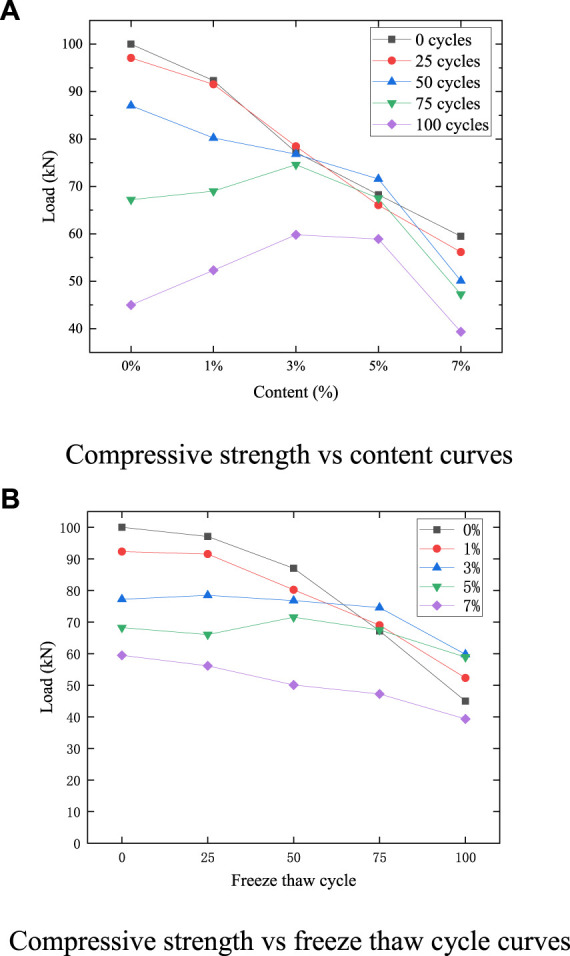
Compressive strength. **(A)** Compressive strength vs. content curves **(B)** Compressive strength vs. freeze thaw cycle curves

Figure 2 shows the effect of microcapsules and freeze-thaw cycles on the frost resistance of cement-based composites. Figure 2A shows the change of compressive strength of each content after different freeze-thaw cycles. By observing the curves of 0, 25, and 50 cycles, it can be found that the compressive strength decreases with the increase of the content of microcapsules, indicating that the microcapsule has not taken effect at this time, and the defect effect itself still plays a major role. The compressive strength of 50 cycles at the content of 1%, 3%, and 5% has a low decline and is relatively flat. This is because, with the increase of microcapsules, the self-healing effect begins to appear. At this time, the enhancement effect brought by microcapsules has exceeded the defect effect. The reason for this phenomenon is that the microcapsules distributed on the surface are easy to be triggered at the same time as freeze-thaw damage, which can be proved by a large number of damaged microcapsules on the surface of the specimen. During the melting period, the microcapsule reacts with water, which can not only repair the cracks, but also strengthen the interior of the specimen, making it not easy to crack and peel off, and increasing the frost resistance. With the increase in the number of cycles, this law began to become more and more obvious. After 75 and 100 cycles, the compressive strength first increased and then decreased, indicating that the healing effect of an appropriate amount of microcapsules such as 3% and 5% will greatly exceed the defect effect and enhance the frost resistance.

Figure 2B shows the changes in the compressive strength of cement-based composites with different microcapsule contents and different freeze-thaw cycles. It can be found that the compressive strength of 0%, 1%, and 7% content decreases with the increase of freeze-thaw cycles, but the reduction rate of 7% content is significantly lower than that of 0% and 1%. This means that although the compressive strength of 7% content is low, it also plays a certain role in strengthening. The compressive strength of 0% content is slightly higher than 1%, but after 75 and 100 freeze-thaw cycles, the compressive strength of 1% content exceeds 0%, indicating that the microcapsule plays a role at this time. Although the compressive strength of 3% content mixed in 0, 25, 50, and 75 cycles decreases slightly, it is almost identical, which indicates that the frost resistance of the specimen is enhanced. The compressive strength of 5% content decreases first and then increases, and the compressive strength is slightly lower than 3% content after 100 cycles. After 100 cycles, the strength of 3% content is the highest, followed by 5% content, 1% content, 0% content and 7% content.

In general, the addition of an appropriate amount of microcapsules will improve its frost resistance, with a slight increase of 1% content, 3% content, and 5% content. Although the reduction rate of 7% content is slow, which reflects the certain enhancement of frost resistance, due to the excessive introduction of defects, the overall compressive strength is low, which is not suitable for practical engineering application.

### AE characteristics

**FIGURE 3:**
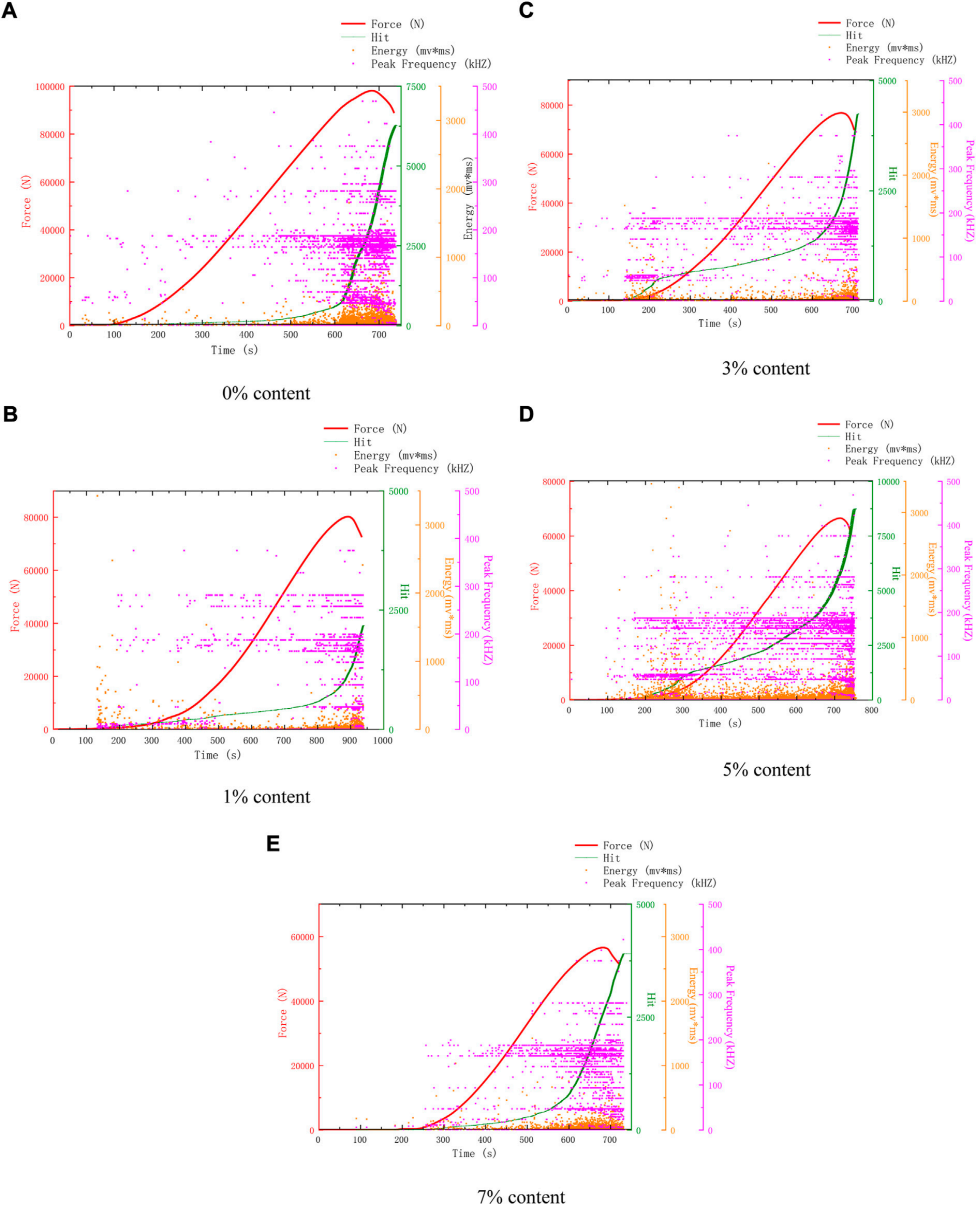
AE characteristics of samples after 25 freeze-thaw cycles. **(A)** 0% Content **(B)** 3% Content **(C)** 1% Content **(D)** 5% Content **(E)** 7% Content.

Figure 3 shows the AE characteristics of compression failure of specimens with different microcapsule contents after 25 freeze-thaw cycles. AE characteristics can reflect the compactness, the change of internal holes, and the trigger of microcapsules to a certain extent. This paper mainly uses the change of AE hits to study the freeze-thaw failure and self-healing mechanism of self-healing cement-based composites. Compared with 25 freeze-thaw cycles and 0 freeze-thaw cycles in Figure 3, the acoustic emission count of 0% self-healing cement-based composites increased, and the compressive strength decreased slightly. This is because the freeze-thaw cycle will cause slight peeling to the surface and pore development. Therefore, it is clear that the damaging effect caused by 25 freeze-thaw cycles is low. The strength of 25 cycles and 0 cycles decreases with the increase of contents. The AE characteristic curves of 0% and 1% are similar, but the difference is that the AE hits of 1% are lower than that of 0%.

Observing the AE characteristic curves of 3%, 5%, and 7%, it can be found that the AE events in the whole process become dense. Different from 0 freeze-thaw cycles, the AE hits of 5% content are higher than 3% content, and the intensity is still lower than 3% content. The main reason is that more microcapsules are triggered.

The AE hits and intensity of 7% are greater than 0 cycles. This is because the microcapsules on the outer surface are damaged by freeze-thaw and are easy to trigger. The expansion prevented further deterioration, and the repair further strengthened the specimen. The expansion of microcapsules and the enhancement of compressive strength lead to the increase of AE hits. The compressive strength of some samples even exceeds that of the original samples. The reason is that the self-healing effect of microcapsules after triggering is greater than that of freeze-thaw damage and introduced defects, which means that it is practical to use self-healing microcapsules to improve frost resistance.

**FIGURE 4:**
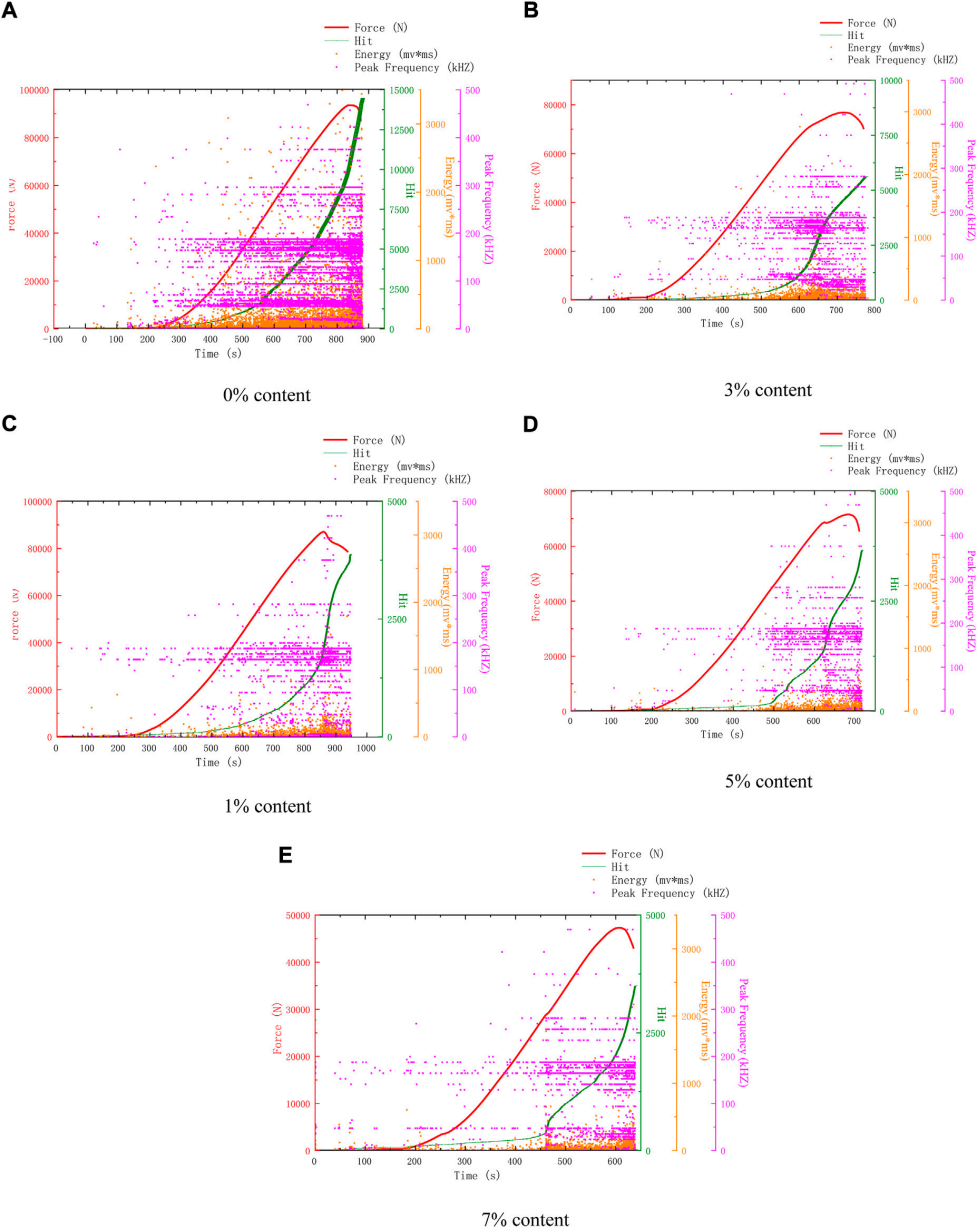
AE characteristics of samples after 50 freeze-thaw cycles. **(A)** 0% Content **(B)** 3% Content **(C)** 1% Content **(D)** 5% Content **(E)** 7% Content.

Figure 4 shows the AE characteristics of compression failure of specimens with different microcapsule contents after 50 freeze-thaw cycles. Compared with 25 freeze-thaw cycles, the compressive strength of 0% content in 50 freeze-thaw cycles decreased slightly, but the AE hits increased sharply. It can be seen that after 50 freeze-thaw cycles, its surface and interior were damaged. The surface is eroded and damaged, loose and easy to peel off, and the internal voids are developed, resulting in the increase of AE hits. The AE hits of the remaining dosage were lower than 0%, and the healing and reinforcement of microcapsules began to play a role, inhibiting the surface spalling and the development of internal voids, making the surface relatively complete and the interior relatively dense.

**FIGURE 5:**
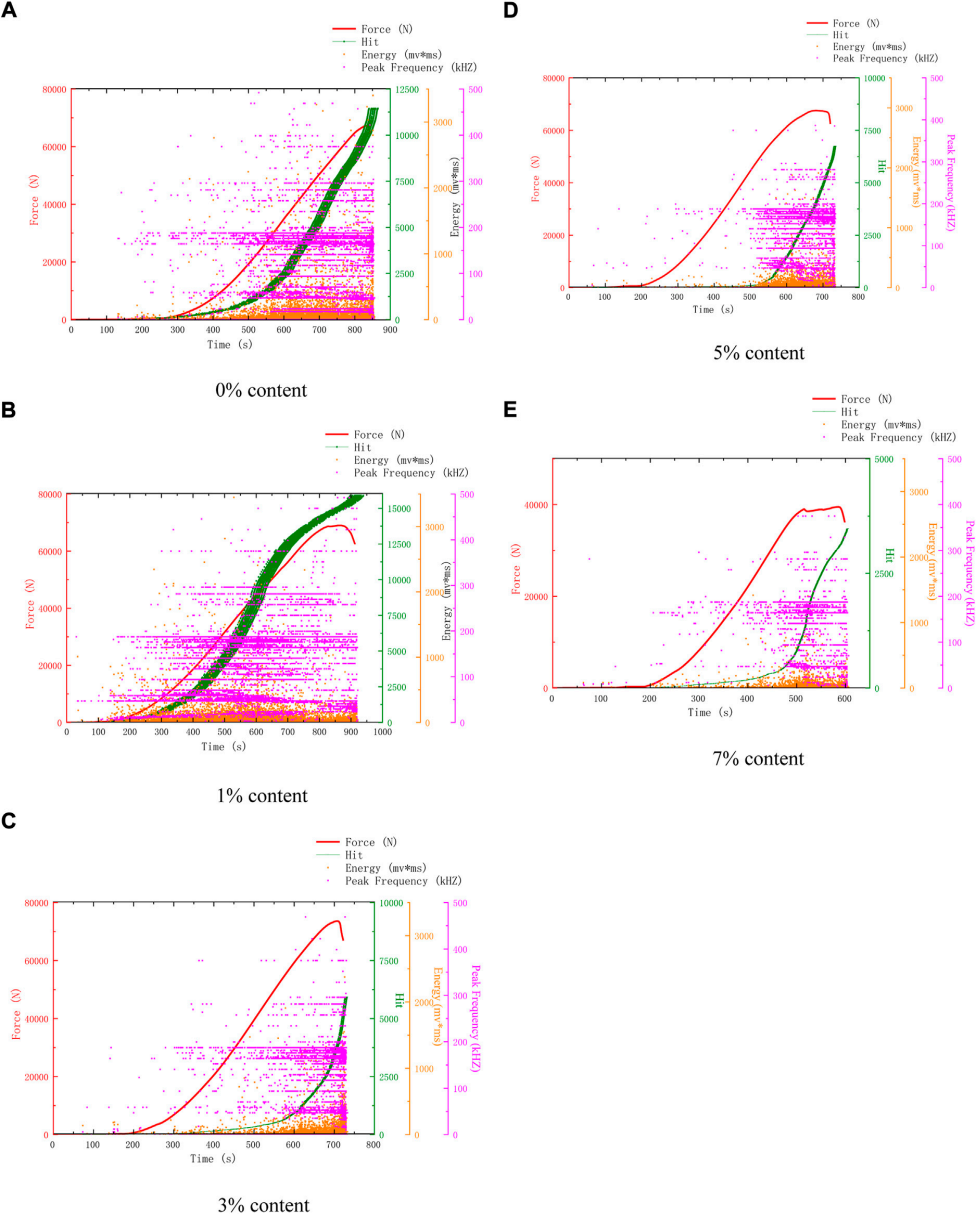
AE characteristics of samples after 75 freeze-thaw cycles. **(A)** 0% Content **(B)** 1% Content **(C)** 3% Content **(D)** 5% Content **(E)** 7% Content.

Figure 5 shows the AE characteristics of compression failure of specimens with different microcapsule contents after 75 freeze-thaw cycles. By observing Figure 5, the AE characteristic curve can be divided into two types. The first type is 0% and 1% content, which has a large number of AE events and dense distribution in the whole process. The second is 3%, 5%, and 7%. The AE events are less in the early stage and begin to increase in the middle and late stages. The decrease in previous events means that microcapsules play a role in repair, and their pore structure becomes dense.

**FIGURE 6:**
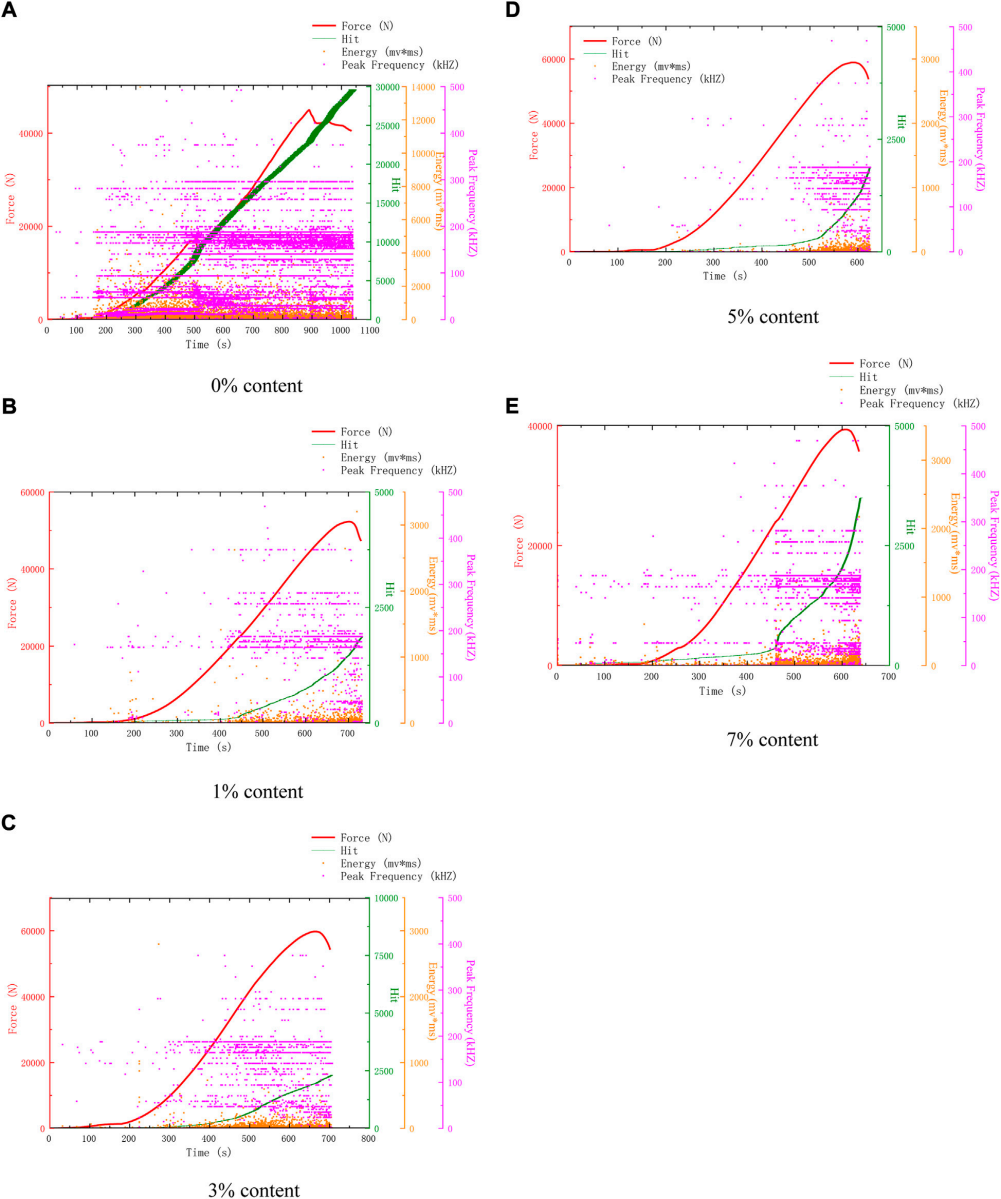
AE characteristics of samples after 100 freeze-thaw cycles.

**FIGURE 7:**
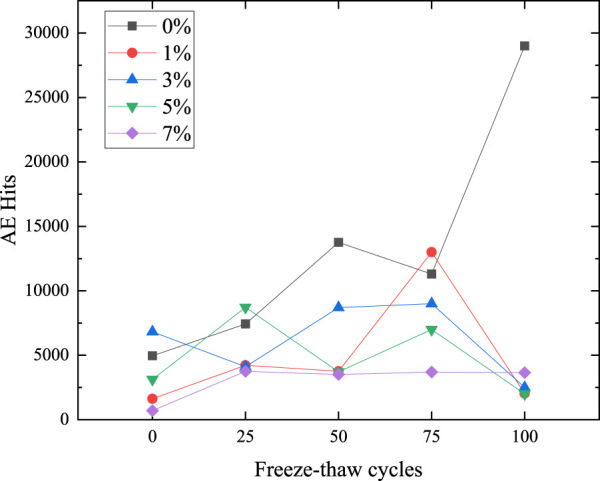
AE hits.

Figure 6 shows the AE characteristics of compression failure of specimens with different microcapsule contents after 100 freeze-thaw cycles. The change in the AE characteristic is affected by many factors. Concrete strength, random distribution of microcapsules, different internal temperature of the freeze-thaw circulator, and random fracture of cement-based composites may affect the AE characteristic. Therefore, we should not only compare the details, but also analyze the AE characteristic as a whole. From Figure 3 to Figure 6, some typical features are selected for analysis. Finally, the AE hits of each content are shown in Figure 7. The coupling of various factors leads to the fluctuation of the details, but it is found that it has a certain law from the overall analysis. The AE hits of 0% content increase and the compressive strength decrease, indicating that the 0% content specimen is deteriorating and not restrained. The AE hits of 1% dosage are lower than 0% dosage in almost every cycle, indicating that the addition of microcapsules plays a certain role in healing and protection. However, after 75 freeze-thaw cycles, the AE hits is close to or even more than 0% of the sample, which indicates that the protective effect of 1% begins to weaken after 75 freeze-thaw cycles. Before 50 freeze-thaw cycles, the AE hits of 3% content was more than that of 5%, but changed after 50 cycles. Combined with the fact that the intensity of 5% content was higher than that of 3% content after 75 cycles, it can be inferred that the internal structure of 5% content was better than that of 3% content after 75 cycles.

Because 5% content has a good healing effect, according to the changes in surface and AE parameters, the healing process is divided into pre-healing and post-healing. In the early stage of healing, a large number of microcapsules can be observed on the surface due to the high content of 5%. After 25 cycles, a large number of white spots can be found, which are the triggered microcapsules. But at this time, because the healing time is short, it is easy to fall off, and the bonding effect has not yet played a role. At this time, there is a gap between the microcapsule and the matrix, because the microcapsule is not triggered completely and is not integrated with the structure, so there are many AE events. In the later stage of healing, after 50 cycles, it can be seen that the spacing around a large number of microcapsules no longer exists. At this time, they have been integrated as a whole, and the texture is hard and not easy to peel off. The surface of the 0% content specimen is mostly loose sand particles after peeling, which is easy to fall off when touched by hand. There are many AE events when it is damaged by compression. 3%, 5% surface peeling is reduced, and the texture is hard and not easy to fall off. There is a very obvious trend of 5% content. After 25 cycles, AE events are all over the whole process, as shown in Figure 3D. However, after 25 cycles, the early AE events become less, as shown in Figure 4D, Figure 5D, and Figure 6D. Compared with the situation of more AE events in the whole process of 0% content shown in Figure 4A, Figure 5A and Figure 6A, it shows that its pore structure is improving.

The AE hits in the whole process of 7% content increases first and then fluctuates very little, which can be explained from the aspect of strength. The healing effect of microcapsules can reduce AE events, and freeze-thaw damage will increase events. The increase is due to the increase in the strength of the specimen due to the triggering of the microcapsule after 25 cycles. However, after 50 cycles, the strength is almost unchanged, because the microcapsules will be continuously triggered from the outside and inside, and the freeze-thaw damage also continues to occur. The effect of continuous trigger healing offsets the impact of freeze-thaw damage. Due to its large defects, although it has a good healing effect, the strength has always been low.

Except that the AE hits of 0% content have been increasing, the AE events of other dosages began to decrease after100 cycles, because after 100 cycles, it not only triggered more microcapsules, but also healed for a longer time. Its adhesion fixed the matrix and improved the pore structure. At the same time, the compressive strength is reduced, and the damage occurs soon, the compression time is reduced, and the AE events are reduced.

### Mass loss rate

When the curing age of the cement-based composite specimen reaches 28 days, take out the specimen and test its initial quality Wft0i. Then, according to the experimental design, the mass of the specimens under different freeze-thaw cycles was measured (Wftni). Calculate the mass loss rate of the test piece after the freeze-thaw cycles through Eq. 1.

$$\Delta Wftni=\frac{Wft0i-Wftni}{Wft0i}\times 100\%$$ (1)

**FIGURE 8:**
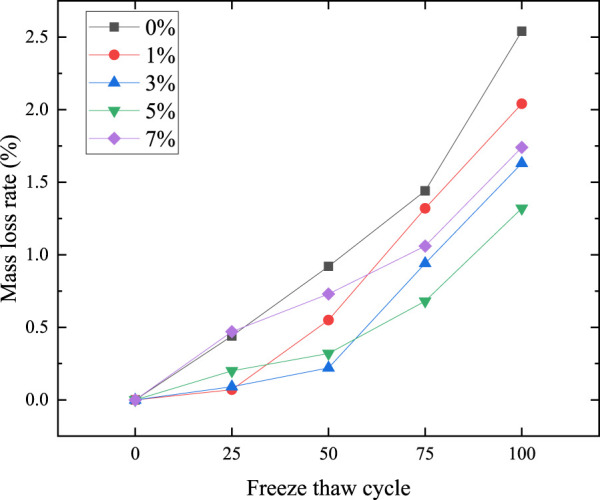
Mass loss rate of each freeze-thaw cycle.

Figure 8 shows the mass loss rate of microcapsule cement-based composites under different freeze-thaw cycles. After 25 freeze-thaw cycles, the mass loss rate of 1% content is the lowest, only 0.07%. The reason is that the low content of sodium fluosilicate improves the pore structure, and the microcapsules also have a certain repair and protection effect. The mass loss of 0% and 7% samples is the most, which are 0.44% and 0.47% respectively, and the mass loss rate of 7% samples is slightly higher than that of 0%. The reasons for the two kinds of high loss are different. The 0% content specimen is because the damage suppression measures are not taken. The 7% content specimen is due to the addition of excessive microcapsules, which makes the microcapsules easy to agglomerate on the surface and weaken the adhesion with the matrix. Moreover, after the freeze-thaw cycles, the external microcapsules are easy to fall off as a whole. In addition, excessive microcapsules will also deteriorate the internal structure.

After 50 cycles, the mass loss rate of the specimen with 1% content is 0.55%, second only to 0.92% of 0% content and 0.73% of 7% content, which means that it still has a good protective effect at this time, but the effect is reduced due to the small content. The surfaces of 7% content specimens are easy to fall off, and the microcapsules embedded in the matrix begin to play a role and inhibit the mass damage. At this time, the sample with the least mass loss is the sample with 3% content, which is only 0.22%.

After 75 and 100 cycles, the mass loss rate of the 5% sample is the lowest, which is 0.68% and 1.32% respectively, which means that it has a good protective effect. The protection effect of 1% and 3% samples is better in the early stage. Although the test piece with 7% content reflects a certain effect, the quality loss rate has been at a high level because of its large defects.

The overall observation shows that the mass loss rate of 0% content is always high, while the sample with microcapsules is low, which verifies the conclusion that microcapsules can inhibit mass loss. After 100 freeze-thaw cycles, the lowest mass loss rate of specimens with 5% content is 1.32%, which is far less than 2.54% of 5% content.

### Experimental results of electric flux

The electric flux method is a widely used test method to evaluate the chloride penetration resistance of cement-based composites. Porosity is the key factor affecting the chloride permeability of cement-based composites. Many scholars have found that there is a great correlation between the chloride permeability of cement-based composites and the porosity of slurry. Generally speaking, the greater the porosity of cement-based composites, the lower the compactness and chloride ion penetration resistance of cement-based composites, and the higher the electric flux.

**FIGURE 9:**
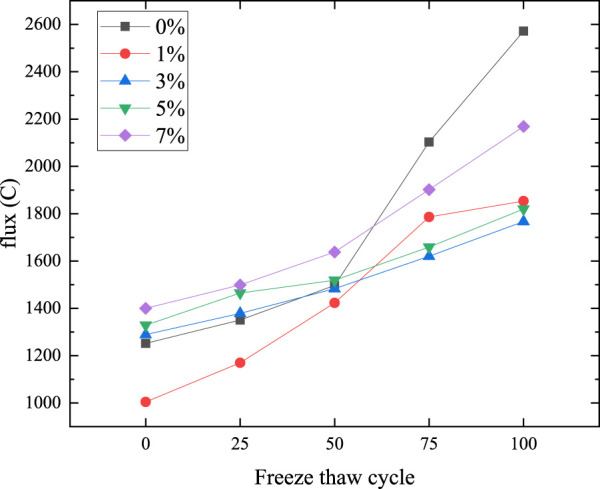
Electric flux after different freeze-thaw cycles.

The permeability coefficient of chloride ions reflects the pore state to some extent. The study of electric flux can reflect the permeability and thus the effect of self-healing. Figure 9 shows the electric flux of microcapsule self-healing composite samples with different contents after different freeze-thaw cycles. When the freeze-thaw cycle is not carried out, the 1% content test block has the best impermeability, because sodium silicate and its curing agent sodium fluosilicate have certain impermeability, and a small amount of addition can make up for its own defects. With the increase of content, the defects brought by it are larger and larger, and the experimental results show that the electric flux is larger and larger. The increased permeability may be due to the poor compatibility between cellulose shells and cement-based, resulting in more pores. Also because of the addition of microcapsules, the volume of cement and the proportion of cementitious body are relatively reduced. With the increase of the content of microcapsules, the capsule particles will produce the so-called crowding phenomenon. The capsule particles will hinder the movement of the surrounding fluid and increase the viscosity of the mortar (Cui et al., 2020; Liu et al., 2020), resulting in poor compaction of the test piece and reducing the permeability of the material.

With the increase of freeze-thaw cycles, the electric flux increases slightly, and the law remains unchanged when 25 freeze-thaw cycles are carried out. When 50 cycles are carried out, the 1% content sample still has well anti permeability. At this time, the electric flux of 0%, 3%, and 5% content samples are very close, but the electric flux of 3% content has been lower than 0%, and that of the 5% content is slightly higher than 0%, indicating that the 3% content anti permeability has been slightly higher than 0%, and the 5% content anti permeability is still lower than 0%. When 75 and 100 cycles are carried out, the electric flux of 0% content has been significantly higher than that of other contents. The 7% content is too large because of its own defects, and the electric flux is second only lower than 0%. At this time, the electric flux of 1% content is higher than 3% and 5%, which is because the number of microcapsules is small, and the ability of self-healing is almost lost in the later stage.

As the control group, the 0% content test block has better impermeability in the early stage, only lower than the 1% test block, but after 75 cycles, the impermeability is the worst because there is no self-healing effect of microcapsules. 1% has the best impermeability before 50 cycles, because sodium silicate and its curing agent sodium fluosilicate have certain impermeability, and a small amount of addition can make up for its own defects. The effect weakened after 75 cycles, because the number of microcapsules was small, and the ability of self-healing was almost lost in the later stage. The content of 3% is similar to that of 5%. The impermeability effect is general before 50 freeze-thaw cycles, but it shows good impermeability in the later stage. The impermeability effect of 3% is slightly better than that of 5%. The reason may be that the healing effect of 3% is higher than that of defects, but the defect effect after 5% is greater than that of healing. The impermeability of 7% has been the worst before 75 cycles, and the defect effect is greater than the healing effect, but the impermeability effect is better than 0% after 75 cycles, which indicates that the self-healing microcapsule also has a certain effect.

## Conclusion

In this paper, the changes in AE characteristics, compressive strength, mass loss rate, and electric flux of microcapsule self-healing cement-based composites after freeze-thaw cycles were studied, and the relationship between microcapsule contents and these parameters was discussed. The main conclusions are as follows:

(1) Because of the different triggering methods, the AE characteristics of compression failure can better reflect the internal changes in the whole process. The influence of microcapsules on the freeze-thaw failure of cement-based composites from the AE hits and the frequency of AE events in three periods combined with the compressive strength were analyzed.
(2) Because of the different contents of microcapsules, the improvement effect and defect effect change dynamically with the number of freeze-thaw cycles, which is reflected in the dynamic process of strength change.
(3) An appropriate amount of microcapsules can enhance frost resistance. The self-healing and frost resistance provided by microcapsules are greater than the defects brought by themselves, which is finally reflected in the strength change and mass loss of specimens.

## Data Availability

The raw data supporting the conclusion of this article will be made available by the authors, without undue reservation.
